# In situ treatment with a TLR9 agonist virus-like particle to promote immune responses against oral epithelial dysplasia progression

**DOI:** 10.1007/s00262-025-04023-1

**Published:** 2025-05-03

**Authors:** Yan Xu, Alexandra Mcmillan, Nikesh Gupta, Caitlin D. Lemke-Miltner, Aseel O. Rataan, Sudartip Areecheewakul, Divya S. Bhat, Emily A. Lanzel, Sean M. Geary, Andrean L. Simons, George J. Weiner, Aliasger K. Salem

**Affiliations:** 1 Department of Pharmaceutical Sciences and Experimental Therapeutics, College of Pharmacy, University of Iowa, Iowa City, IA USA; 2 Department of Otolaryngology, University of Iowa Hospitals and Clinics, Iowa City, IA USA; 3 Holden Comprehensive Cancer Center, The University of Iowa, Iowa City, IA USA; 4 Department of Internal Medicine, The University of Iowa, Iowa City, IA USA; 5 Department of Oral Pathology, Radiology and Medicine, College of Dentistry, University of Iowa, Iowa City, IA USA; 6 Department of Pathology, University of Iowa, Iowa City, IA USA; 7 Department of Clinical Pharmacy and Pharmacy Practice, Faculty of Pharmacy, Yarmouk University, Irbid, Jordan; 8 Department of Pharmacology and Physiology, Faculty of Pharmaceutical Sciences, Chulalongkorn University, Bangkok, Thailand; 9 Department of Radiation Oncology, Carver College of Medicine, Iowa City, United States

**Keywords:** Oral epithelial dysplasia, Immunotherapy, Virus-like particle, Plasmacytoid dendritic cells

## Abstract

Leukoplakia, a common type of oral dysplasia, is simply defined as a white patch in the mouth or other mucosal surface. Oral dysplasia is the most common premalignancy in the oral cavity and yet it is insufficiently researched and thus both diagnosing and treating oral dysplasia are still problematic issues. This study focuses on the immune signature of oral dysplasia and explores whether stimulating the immune system with an immune therapy, vidutolimod (± immune checkpoint blockade (ICB)), can prevent the progression of oral dysplasia or even cause regression. Vidutolimod, a virus-like particle encapsulating G10, is believed to activate plasmacytoid dendritic cells (pDCs) through the activation of the Toll-like receptor 9 (TLR9). To investigate this, an established murine model for inducing oral cancer was used to study oral dysplasia development and response to in situ injection of vidutolimod at the premalignant phase. The effect of treatment was analyzed histologically and immunologically. ELISA revealed significantly elevated levels of IFN-γ, IL-12, and TNF-α in the sera of mice after 24 h of one treatment with vidutolimod + ICB as well as increased levels of proliferating T cells and pDCs in draining lymph nodes 72 h after the third and final treatment, thus indicating the immune-boosting effect of this therapy. Vidutolimod + ICB caused a significant decrease in Ki-67 expression by epithelial cells in the lesion area compared to untreated mice, implicating that this treatment regime may prevent lesion progression.

## Introduction

Oral dysplasia is the most common premalignancy in the oral cavity; characterized by white patches inside the mouth which comprise thickened keratin layers of the oral epithelium that are not present in healthy individuals [1]. The precise etiology of oral dysplasia is unclear; however, its occurrence has been correlated with chronic irritation from tobacco use, imbibing alcohol, or chewing betel nuts [2]. Based on data published from 1984 to 2002, the worldwide prevalence of oral dysplasia is approximately 1.49% to 2.60% and the majority of oral squamous cell carcinomas (OSCCs) arise from oral dysplasia which presented as a leukoplakia [3]. However, leukoplakia is still insufficiently researched and both diagnosing and treating oral dysplasia presenting as leukoplakia are still problematic issues in need of being addressed.

Clinical diagnosis of oral dysplasia is achieved through the exclusion of any other possible diseases that could also cause white patches or plaques in the oral cavity, followed by biopsy analysis by pathologists for confirmation of oral dysplasia based on architectural and cellular changes in tissue (Table 1) [4]. Once diagnosed, the disease is then categorized according to severity [3]. The two most common ways of categorizing oral dysplasia are the classification by the world health organization (WHO) in 2017 (mild/moderate/severe) [5] and the binary system published by Kujan et al. [6] in 2006 (low/high risk of developing into cancer) [7]. However, there are no biomarkers that are specific for oral dysplasia, which means diagnoses and categorizing by either system are highly reliant on the judgment made by the pathologists according to cellular and architectural phenotypes.

**Table 1:** Diagnosis criteria for oral dysplasia; diagnosis of these oral dysplasia biopsies was based on cellular and architectural differences

Cellular	Architectural
Anisonucleosis and pleomorphism	Loss of polarity
Anisocytosis and pleomorphism	Hyperkeratosis & parakeratosis & dyskeratosis
Hyperchromatic nuclei	Increased cellular density
Abnormal mitotic figures	Bulbous drop-shaped rete pegs

Current treatments for oral dysplasia include conservative drug therapies and surgical removal; otherwise watchful waiting is implemented. Watchful waiting is a common strategy, particularly when dealing with mild oral dysplasia since such lesions can resolve without any treatment once exposure to the chronic irritation is stopped. However, this strategy carries the risk of further progression of oral dysplasia and even transformation to malignancy. Conservative drug treatments include vitamin A derivatives (e.g., retinoids such as isotretinoin) and precursors of vitamin A (e.g., *β* carotene) [8–10]. However, the high rate of relapses after conservative treatment, combined with toxicity issues and limited efficacy are the main problems with these drugs. Surgical removal with narrow margins (for mild oral dysplasia) still carries the risk of malignant transformation, while aggressive surgical removal (for severe oral dysplasia) has a high recurrence rate (up to 34.7%) and high malignant transformation rate (up to 17.8%) [11]. Thus, further understanding of, and effective therapy for, oral dysplasia are still highly desirable goals in order to lower the risk of recurrence and malignant transformation of oral dysplasia.

Immunotherapy is a treatment strategy that helps boost a patient’s immune system against cancer, which has achieved promising results in a wide range of cancers [12–14]. Building on the success of immunotherapy, a strategy called immunoprevention was introduced by stimulating the body’s immune system to target precancerous cells or early-stage cancer to prevent cancer development [15]. It has been found that there are more infiltrating immune cells (e.g., CD8 + T cells, CD4 + T cells) in oral tissues with high-grade stages of oral dysplasia compared with low-grade stages of oral dysplasia [16–18], suggesting that immune-based therapies capable of further activating these infiltrating cells could have anti-tumor effects. Toll-like receptors (TLRs) are pattern recognition receptors that identify various pathogen-associated molecular patterns (PAMPs) or danger-associated molecular patterns (DAMPs). When PAMPs or DAMPs bind to TLRs on immature antigen-presenting cells, they can trigger their activation and maturation [19]. Toll-like receptor 9 (TLR9), a member of the TLR family, has shown great potential as a target for the treatment of cancer [20], with plasmacytoid dendritic cells (pDCs), constitutively expressing TLR9 being the primary target [21]. After stimulation by a TLR9 agonist, pDCs are activated, triggering a number of downstream effects including the activation NK cells and increasing the expansion of CD8 + T cells [22–24], thereby potentiating anticancer immune effects.

TLR9 signaling can be stimulated by unmethylated cytosine–phosphate–guanine (CpG) oligodeoxynucleotides (ODN) [21]. Among the CpG family, CpG-A is able to activate pDCs and in these studies G10, a CpG-A ODN (G10, gggggggggggacgatcgtcgggggggggg), was used [25]. However, due to the vulnerability of G10 to DNases [26], vidutolimod, a virus-like particle encapsulating G10, was used in these studies, thus protecting G10 from degradation as well as assisting G10 delivery to, and activation of, pDCs [27–29]. A virus-like particle (VLP) is a structure composed of viral proteins but lacks viral genetic material, rendering it noninfectious. This unique characteristic allows VLPs to induce strong immune responses without causing infection. Vidutolimod has shown promising therapeutic effects in combination with ICB against melanoma in a clinical study [30] as well as head and neck squamous cell carcinoma in a murine preclinical study [31]. In many cancer cases, the efficacy of anti-PD1 alone is limited due to the poor immunogenic tumor/lesion microenvironment [30, 32]. Vidutolimod’s ability to promote an inflammatory (“hot”) tumor microenvironment can potentially function synergistically with anti-PD1 to prevent cancer progression. It has been reported that ICB (e.g., anti-PD1) can induce oral dysplasia regression among patients with high-grade oral dysplasia [33]. However, in this clinical trial, only 36% of patients showed the best overall response after treatment, meaning there was a more than 40% decrease in size and degree of dysplasia. Vidutolimod through the activation of pDCs may potentially increase the activity and number of CD8 + lesion-specific T cells in the tumor microenvironment and work synergistically with anti-PD1 [31]. Therefore, a better therapeutic effect may be achieved. Here, we use an immunocompetent oral dysplasia model [34] to test the ability of vidutolimod + ICB to alter the oral dysplasia immune environment and suppress progression of the disease.

## Materials and method

### Spatial immunological analysis of different grades of oral dysplasia

Formalin-fixed paraffin-embedded human oral dysplasia samples were collected from the College of Dentistry, University of Iowa. A 5-µm-thick biopsy was made from different grades of epithelial dysplasia/healthy oral tissue (healthy (*n* = 5), low-grade dysplasia (*n* = 6), high-grade dysplasia (*n* = 11)), and sent to Nanostring Technologies (Seattle, US) for spatial biological analysis. Firstly, samples were stained with imaging reagents for CD4, CD8, PanCK, and DNA. Based on the staining results and lesion severity, regions of interest (ROI) were selected on the slides/biopsies with the cooperation of pathologists. Then, a quantitative immune markers analysis was done with profiling reagents provided by Nanostring Technologies. Briefly, the profiling reagents (a panel of immune-related profiling reagents) were composed of antibodies that were able to bind with the target proteins and oligos linked to the antibody by UV-photocleavable bond. Oligo information was collected after the ROI was exposed to UV to cleave the linkage. The extracted oligo representing antibody expression was analyzed, and the data were obtained from the GeoMx Data Analysis software. Out of the collected data, the housekeeping proteins, histone H3 and S6, were used for normalization.

#### Mouse oral dysplasia model development

Mouse studies were approved and performed according to guidelines established by the University of Iowa Institutional Animal Care and Use Committee. Female C57BL6J mice (6–8 weeks of age) were obtained from The Jackson Laboratory. The carcinogen, 4NQO (stock solution: 5 mg/mL), was prepared by dissolving 4NQO (4-nitroquinoline-N-oxide, 98%, Fisher Scientific) in DMSO (Fisher Chemical) and stored at -20 °C until use. To prepare 4NQO drinking water, the stock solution was added to water supplemented with 1% v/v propylene glycol (VWR life science) at a final concentration of 100 μg/mL. Mice were treated with fresh 4NQO carcinogen drinking water every week for 16 weeks, after which the water supply was switched back to normal water. The lesional status on the tongue and weight of mice were closely monitored during carcinogen water administration.

#### G10/Vidutolimod treatment for mice with oral dysplasia-like signs

**Fig. 1:**
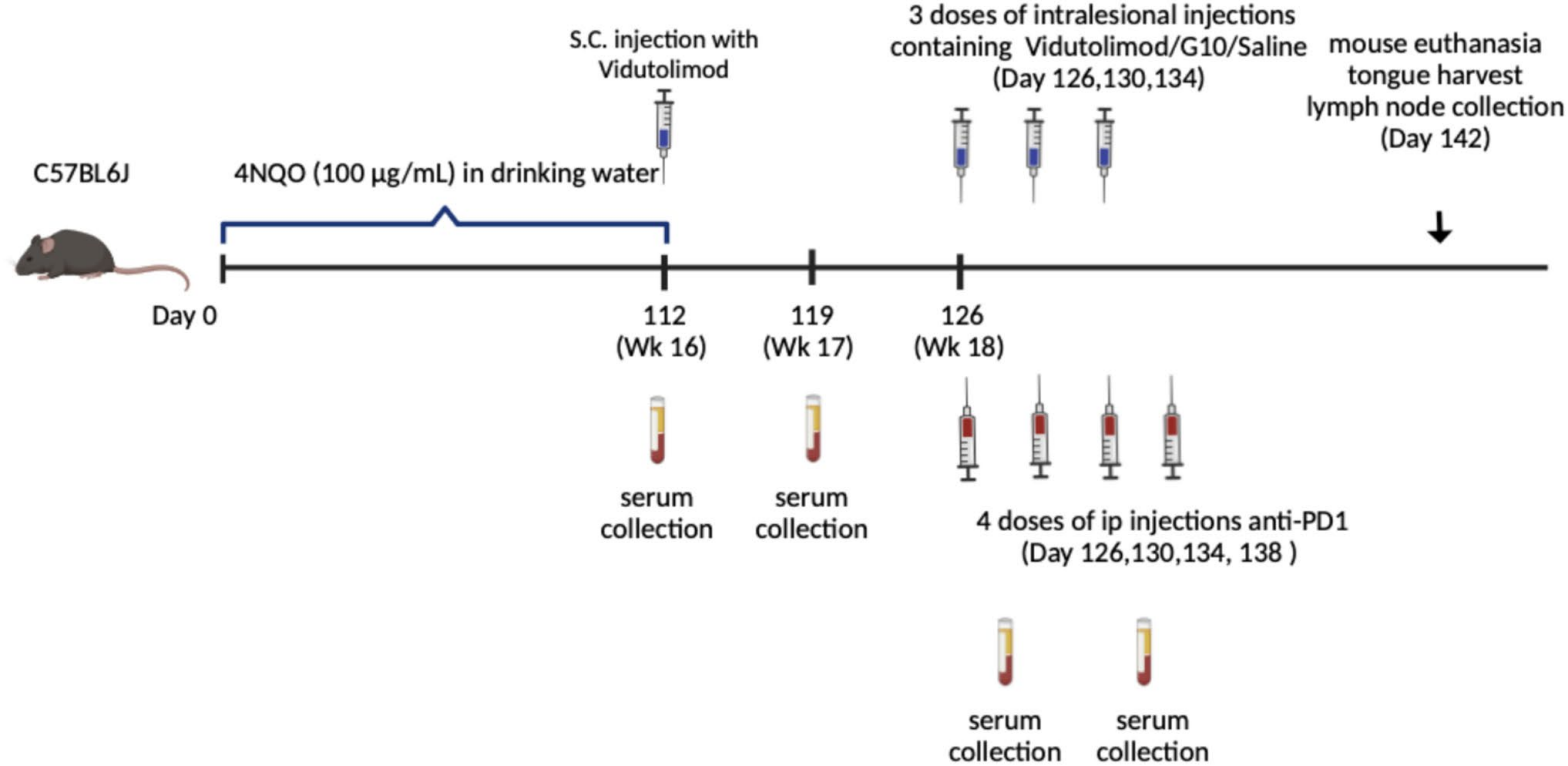
Timeline for 4NQO treatment and vidutolimod treatment

Mice were anesthetized with 100 μL intraperitoneal (IP) ketamine/xylazine before treatment. Vidutolimod was provided by Regeneron (Westchester County, New York). Anti-Qβ IgG (used as a standard for the ELISA to determine anti-Qβ levels in vaccinated/primed mice, see more detail below) and CpG G10 were provided by Dr. Weiner (Carver College of Medicine, The University of Iowa, Iowa, USA) and anti-PD1, clone RMP1-14, was purchased from BioXCell (Lebanon, NH, USA). Mice were divided into five treatment groups (*n* = 5–9/group): 1) saline, 2) G10, 3) vidutolimod, 4) anti-PD1, or 5) vidutolimod + anti-PD1-treated. The appearance of lesions (size and number) was compared between groups before treatment was commenced to ensure all experimental groups had similar grades of oral dysplasia. It was ensured that approximately 50% of mice with high-grade dysplasia were included in each group. To efficiently deliver vidutolimod to pDCs, a priming step is required to facilitate the development of anti-Qβ antibodies. These antibodies specifically bind to vidutolimod, facilitating its uptake by antigen-presenting cells such as pDCs. This approach was validated by Lemke-Miltner et al. [28], demonstrating that vidutolimod significantly activates pDC only in the presence of anti-Qβ. Therefore, all mice were subcutaneously injected with vidutolimod (12.5 μg CpG, 80 μL) two weeks before intralesional injection of treatment to develop anti-Qβ antibody responses (‘prime’). Blood was collected before priming and one week after priming (see Fig. 1 for treatment schematic**)** and the anti-Qβ IgG antibody level was analyzed by ELISA as previously described by Lemke-Miltner et al*.* Treatment started one week after the carcinogen water supply was stopped **(**Fig. 1**)**. Treatments of vidutolimod (100 µg CpG, 80 µL), G10 (100 µg CpG, 80 µL), or saline (80 µL) were administered by intralesional injection every four days for three doses in total. For ICB, anti-PD1 was given by intraperitoneal injection every four days for four doses in total. All mice were sacrificed four days after the last treatment.

#### Mouse draining lymph node analysis by flow cytometry

Single-cell suspensions were made by dissociating harvested medium mandibular lymph nodes using a gentleMACS Dissociator (Miltenyi Biotec). Cells in lymph nodes were stained with Zombie Violet Fixable Viability dye (Biolegend) and washed with FACS buffer (10 g bovine serum albumin (Sigma-Aldrich) into 1 L of PBS with 5 ml 10% (w/v) NaN3 (Sigma-Aldrich)) at 4 °C. Then, cocktails of antibodies against surface markers (CD8 BUV395, CD4 BUV496, Ly6G BUV563, CD19 BUV661, CD11c BUV737 and CD86 BUV805 (BD Biosciences), MHC class ll BV510, Ly6C BV570, CD335 BV605, CD3e BV750, PD1 BV785, CD45 SparkBlue 550, CD11b PE, F4/80 PE-Cy7, B220 APC/FIRE 750, and CDy7 FITC (Biolegend)) were added to samples. TruStain FcX™ (anti-mouse CD16/32) antibody (Biolegend) was added with all surface antibodies as an Fc receptor blocker. For intracellular marker staining, Fix/Perm buffer (Thermos Fisher Scientific) was used to fix and permeabilize cells before adding markers for Ki-67 A700 and FoxP3 Pacific Blue (Biolegend) and cells with antibodies were incubated at 4 °C for 60 min in the dark. UltraComp eBeads (Thermos Fisher Scientific) were used for preparing respective tubes for each antibody. Prepared samples were analyzed by flow cytometry using a Cytek® Aurora Flow Cytometer.

#### Histological and immunohistochemical analysis of potential oral dysplasia on mouse tongues

After mice were euthanatized, their tongues were collected and fixed in 10% non-buffered formaldehyde (NBF) overnight before sectioning. Fixed tongues were dissected vertically at the lesion site. Dissected tongue tissues were transferred to 70% ethanol and embedded in paraffin for histopathologic study. Slides were stained with (i) hematoxylin and eosin (H&E), and (ii) for Ki-67 (rabbit anti-Ki-67 IgG, Abcam) to reveal tumor progression and proliferation, respectively. Stained slides were visualized under microscope BX63. A blind analysis was performed on these H&E slides by two board certified oral pathologists. The oral dysplasia was graded based on the binary system. The region of the lesion area was selected by blind analysis by pathologists for analysis of proliferating (Ki-67 +) cells in that region. The dysplasia grading was initially performed separately. Any disagreements between pathologists resulted in reexamination and came to a consensus upon discussion. Pathologists mapped the area of the lesion and then counting of stained tissue was performed within those mapped out areas.

#### Cytokine changes in mouse serum after priming/treatment

Serum was collected 24 h after first treatment and third treatment. Blood was collected into capillary action blood collection tubes (SAI Infusion Technologies) and clotted at room temperature for 1 h. Serum was collected and kept at -80 °C until analysis. Collected serum was warmed to room temperature and analyzed for IFN-α, IFN-γ, IL-2, IL-4, IL-6, IL-10, IL-12p70, TGF-β 1, and TNF-α with ProcartaPlex multiplex kits (Thermo Fisher Scientific) following manufacturer’s instructions. Then, the plate was read using a BioRad Bio-Plex (Luminex 200). Bio-Plex Manager software was used to calculate concentrations.

#### Statistical analysis

Data are expressed as mean ± SEM. Statistical analysis was performed using GraphPad Prism software for Mac version 10.2.2 (GraphPad Software, Inc., San Diego, CA). Unpaired Student’s t test was performed for nonparametric data. One-way analysis of variance (ANOVA) plus Tukey’s post hoc test was used to compare between three or more groups. Differences were considered significant at *P* < 0.05.

## Results and Discussion

### Immune profiling across patients of different grades of oral dysplasia

**Fig. 2:**
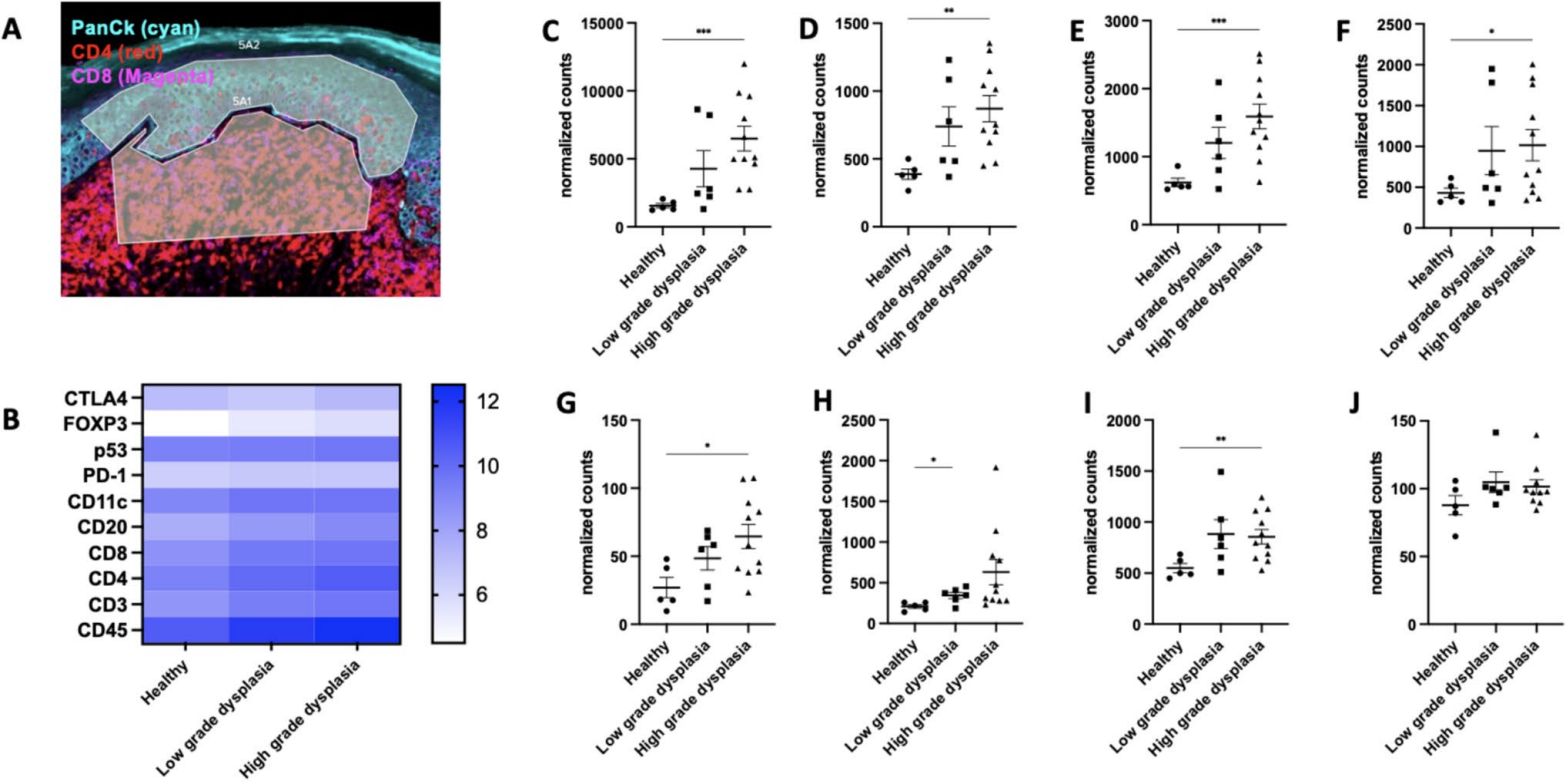
Immune profile of human oral dysplasia sample (connective tissue). **A** ROI selection. The upper region selected is the epithelium; PanCK (pan cytokeratin) was stained with cyan, CD4 + cells stained with red, and CD8 + cells stained with magenta. **B** Heat map of immune cell expression in connective tissue. **C-J** Normalized counts for CD45 + cells, CD3 + cells, CD4 + cells, CD8 + cells, FoxP3 + cells, CD20 + cells, CD11c + cells, PD1 + cells, respectively. The results are expressed as mean ± SEM. Healthy (*n* = 5), low-grade dysplasia (*n* = 6), high-grade dysplasia (*n* = 11). Statistical analysis was carried out by Welch and Brown–Forsythe ANOVA; *, **, *** indicate *p* < 0.05, *p* < 0.01, and *P* < 0.001, respectively. OLP: oral dysplasia

To determine any possible correlation between oral dysplasia progression and immune phenotype, a spatial biologic analysis was done to compare infiltration levels of a range of immune cell types between different grades of human oral dysplasia and healthy oral tissue. Before performing statistical analysis, all data were normalized using the medians calculated from the signal from the housekeeping proteins, histone H3 and S6. It was shown that there was a significantly higher number of CD45, CD3, CD4, CD8, and CD11c positive populations in the connective tissue of the high-risk oral dysplasia patient samples compared with healthy subjects (Fig. 2C-I), which indicated that more leukocytes, such as T cells and DCs, infiltrated into the connective tissue of high-risk oral dysplasia patients. For oral dysplasia biopsies diagnosed as low risk, the difference in immune cell numbers of each tested phenotype between patients and healthy subjects was not statistically significant in the connective tissue (subepithelial zone), although generally larger numbers of CD3, CD8, CD11c, and CD45 positive cells were observed. The significantly greater number of potentially immune effector populations (CD3 + , CD8 + cells) observed in tissue samples from patients with high-risk oral dysplasia suggests that high-risk oral dysplasia may be a better target for immunotherapy than low-risk oral dysplasia. This stems from the observations in other cancer types where tumors considered “hot” or populated with relatively high numbers of effector T cells, are more likely to respond to immunotherapeutic approaches such as ICB. [4]

**Fig. 3:**
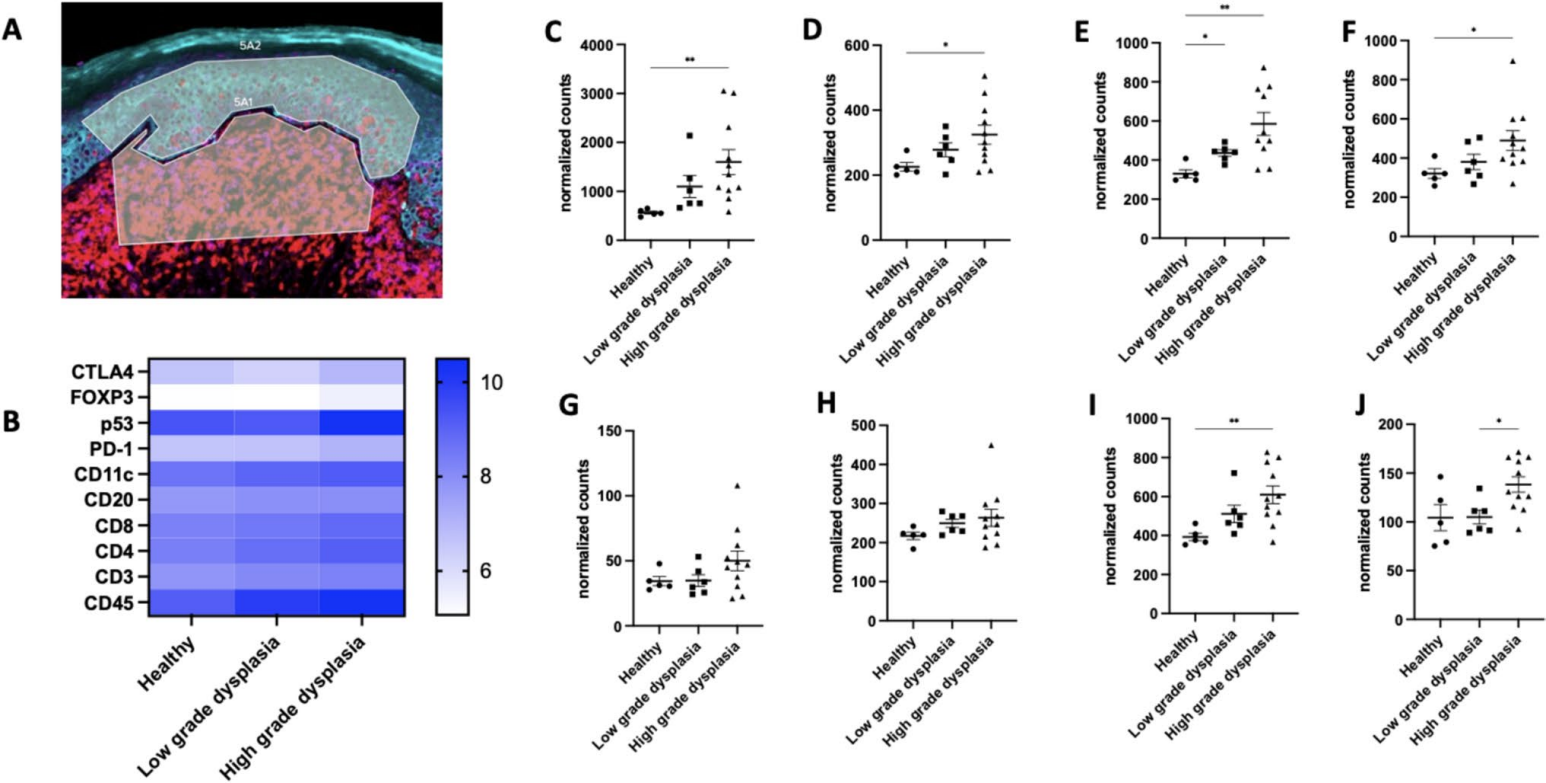
Immune profile of human oral dysplasia sample (epithelium). **A** ROI selection. The region selected below is the connective tissue; PanCK was stained with cyan, CD4 + cells stained with red, and CD8 + cells stained with magenta. **B** Heat map of immune cell expression in connective tissue **C-J** Normalized counts for CD45 + cells, CD3 + cells, CD4 + cells, CD8 + cells, FoxP3 + cells, CD20 + cells, CD11c + cells, PD1 + cells, respectively. The results are expressed as mean ± SEM. Healthy (n = 5), low-grade dysplasia (n = 6), high-grade dysplasia (n = 11). Statistical analysis was carried out by Welch and Brown–Forsythe ANOVA; *, **, *** indicate *p* < 0.05, *p* < 0.01, and *P* < 0.001, respectively. OLP: oral dysplasia

In epithelial ROIs, similar immune profile changes across different grades of dysplasia were found to those observed for the connective tissue ROIs (Fig. 3C-J). In addition, PD1 level was significantly higher when oral dysplasia was severe, which indicates that PD1/PDL1 could be a target for improved immune responses in high-risk oral dysplasia patients (Fig. 3.J). This finding aligns with current research that implicates the PD1/PDL1 pathway as a crucial mediator of immune evasion in various pathologies, including precancerous conditions [35]. Analysis of the human samples indicated that oral dysplasia, particularly at an advanced stage, could benefit from immune therapy such as anti-PD1. To test this hypothesis, a mouse oral dysplasia model was used for testing the capacity of a TLR9 agonist ± anti-PD1 to affect oral dysplasia progression.

### Mouse oral dysplasia model development

**Fig. 4:**
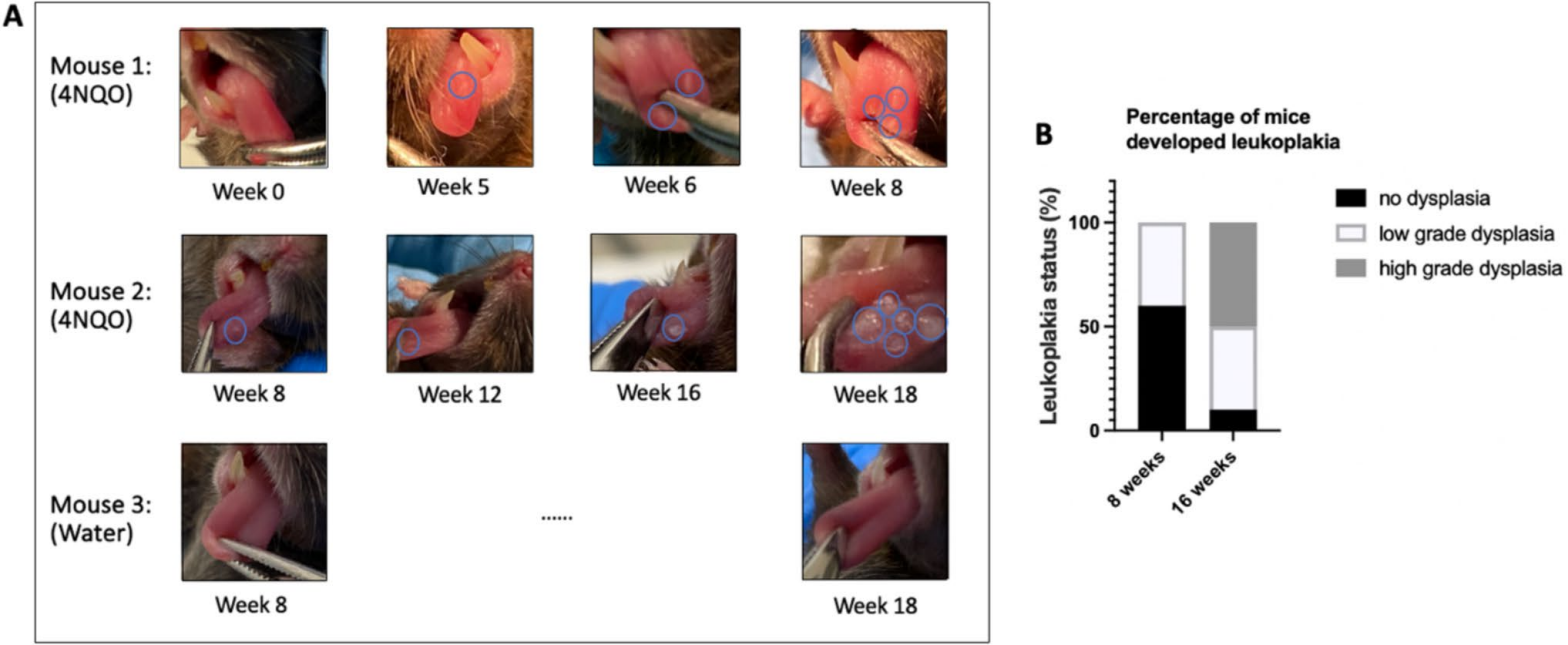
Monitoring a mouse oral dysplasia model. Mouse tongue status after **A** 8 weeks (mouse 1) or 16 weeks (mouse 2) treatment of 4NQO. Lesion size and amount increased throughout 4NQO treatment revealing very mild oral dysplasia at best (lesion area circled). Mouse was treated with normal drinking water. **B** Percentage of mice developing low- or high-grade oral dysplasia after 8- or 16-week 4NQO treatment (n = 10/group)

**Fig. 5:**
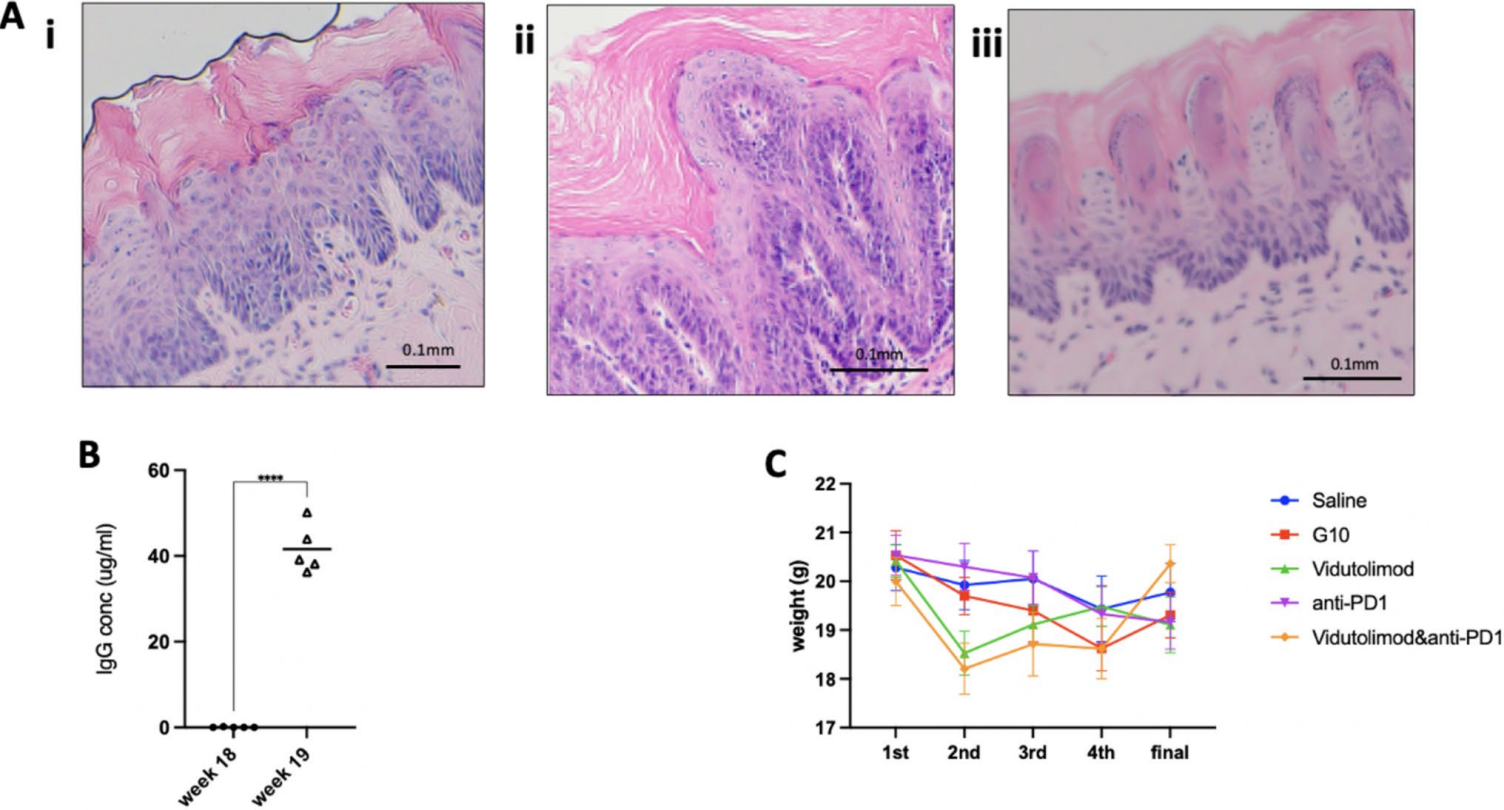
**A** Histological features of different grades of mouse oral dysplasia and healthy mouse tongue. H&E staining of mouse tongues exhibiting: **i** low-grade oral dysplasia after 8-week 4NQO; **ii** high-grade oral dysplasia after 16-week 4NQO; **iii** no oral dysplasia (no 4NQO treatment). **B** Anti-Qβ IgG antibody concentration: before (week 16) and after (week 17) priming. Mouse serum was collected before priming (week 16 of 4NQO treatment) and one week after priming (week 17 of 4NQO treatment) (*n* = 5). **C** Weight change of mice during treatment. First: day of first treatment; second: day of second treatment; third: day of third treatment; fourth: day of fourth treatment; final: day of euthanasia and 4 days after fourth treatment. The results are expressed as mean ± SEM. Treatments were 4 days apart and were commenced 2 weeks after priming with vidutolimod subcutaneously. Unpaired Student’s t test was used for statistical analysis. ** *P* < 0.01

Upon chronic irritation with 4NQO, 8 percent of mice demonstrated white lesions in their oral cavity from week 5, while at the end of week 8, 65 percent of mice had white lesions in their oral cavity (Fig. 4A). After euthanasia, mouse tongues were harvested, formalin-fixed, and paraffin-embedded, then sectioned and stained with H&E. Histopathology slides were sent to pathologists for blind analysis. After 8 weeks of 4NQO treatment, all of the carcinogen-treated tongues possessed thicker keratin layers, which made the tongue look whiter compared with healthy subjects. However, only a limited number of mouse tongues (40%) showed apparent histological differences (e.g., nuclear hyperchromatism, pleomorphism, and increased mitotic figures) versus healthy tongues despite the presence of white lesions, with those 40 percent of mouse tongues being categorized as low-grade oral dysplasia. After 16 weeks of 4NQO treatment, ~ 50 percent of mice developed high-grade oral dysplasia (Fig. 4A & B). Compared to low-grade oral dysplasia (8 weeks) and healthy mice, increased mitotic activity and architectural disorder could be found in high-grade oral dysplasia (16 weeks) (Fig. 5). Consequently, subsequent therapeutic studies (vidutolimod ± anti-PD1) were performed with the model involving 16 weeks of 4NQO treatment (at least ~ 50% of mice exhibited high-grade dysplasia in each group) as this more closely represents a model of high-grade oral dysplasia mimicking human high-grade oral dysplasia features.

### Effect of vidutolimod ± anti-PD1 on mouse oral dysplasia status and histological features

Mice were primed with a single subcutaneous dose of vidutolimod two weeks before intralesional treatment started in order to induce anti-Qβ antibody production. The importance of priming with vidutolimod has been demonstrated previously in an oral cancer model [31]. Serum was collected immediately before priming and one week after priming in order to assess anti-Qβ antibody levels using an ELISA. The results showed that the anti-Qβ antibody levels significantly increased one week after priming (Fig. 5B), which indicates that the mouse immune system possessed the ability to efficiently uptake future administrations of vidutolimod via Fc receptor-mediated endocytosis which is necessary for vidutolimod to induce pDC cytokine secretion [31].

**Fig. 6:**
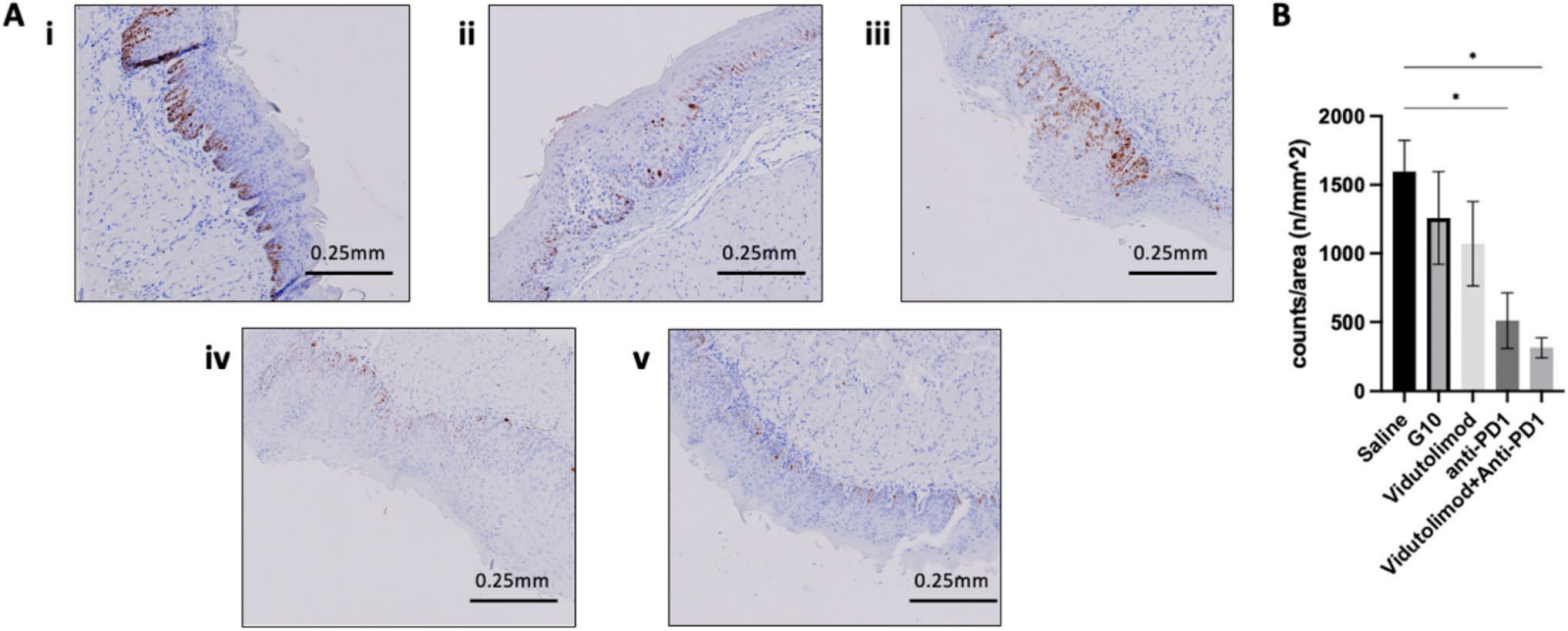
Ki-67 IHC staining analysis. Ki-67 staining result for: **A** **i** saline-treated group (*n* = 7), **ii** G10-treated group (*n* = 6), **iii** vidu-treated group (*n* = 3), **iv** anti-PD1-treated group (*n* = 6), and **v** vidu + anti-PD1-treated group (*n* = 4). **B** Comparison of Ki-67 + cells per lesion area between groups. The results are expressed as mean ± SEM. Vidu: vidutolimod. One-way ANOVA was used for statistical analysis. * *P* < 0.05

Mice were intralesionally injected with: saline, G10, vidutolimod, anti-PD1, or vidu + anti-PD1 (*see *Fig. 1* for treatment regime*). Weight loss data revealed up to a 10% drop in weight after the first treatment for the groups treated with either vidutolimod alone or vidutolimod + anti-PD1; however, these mice started gaining weight back subsequent to the second treatment (Fig. 5C). Due to the limitation of the small lesion size and unreachable locations in the oral cavity, a quantitative analysis could not be applied for the analysis of lesion size. Instead, cell proliferation status was assessed at lesion sites (i.e., sites of epithelial high-grade dysplasia) in mouse tongues collected three days after treatment was completed. Pathologists blindly analyzed IHC slides (stained for Ki-67) and counterstained with hematoxylin to select the lesion area on the tongue. In those lesion areas, there were significantly fewer proliferating (Ki-67 +) cells after anti-PD1 or vidutolimod + anti-PD1 treatment compared to saline-treated controls (Fig. 6). This marked decrease in Ki-67 + ve cells highlights the potential of these agents to suppress tumor progression since Ki-67 expression has been reported to correlate with the severity of oral dysplasia [36]. However, there was no significant difference between the anti-PD1 and the vidutolimod + anti-PD1-treated group in hindering cell proliferation (Fig. 6). The lack of difference between Ki-67 expression in G10/vidutolimod-treated mouse tongue with the control group indicated that TLR9 agonist alone did not efficiently hinder proliferation in the oral dysplasia lesion area. While Ki-67 is widely used in oral dysplasia diagnosis, its limitations—including lack of specificity (as it is expressed in all proliferating cells) and its detection in some non-proliferating mammalian cells—has the potential to compromise its consistency and reliability as a marker for malignancy progression. Therefore, the use of more specific proliferation indicators such as BrdU may be of additional benefit in future studies. Aside from the differences observed with respect to Ki-67 staining, morphologic or architectural differences as defined by H&E staining could not be discerned between treatment groups.

### Effect of vidutolimod ± anti-PD1 on immune cell infiltration to draining lymph nodes

**Fig. 7:**
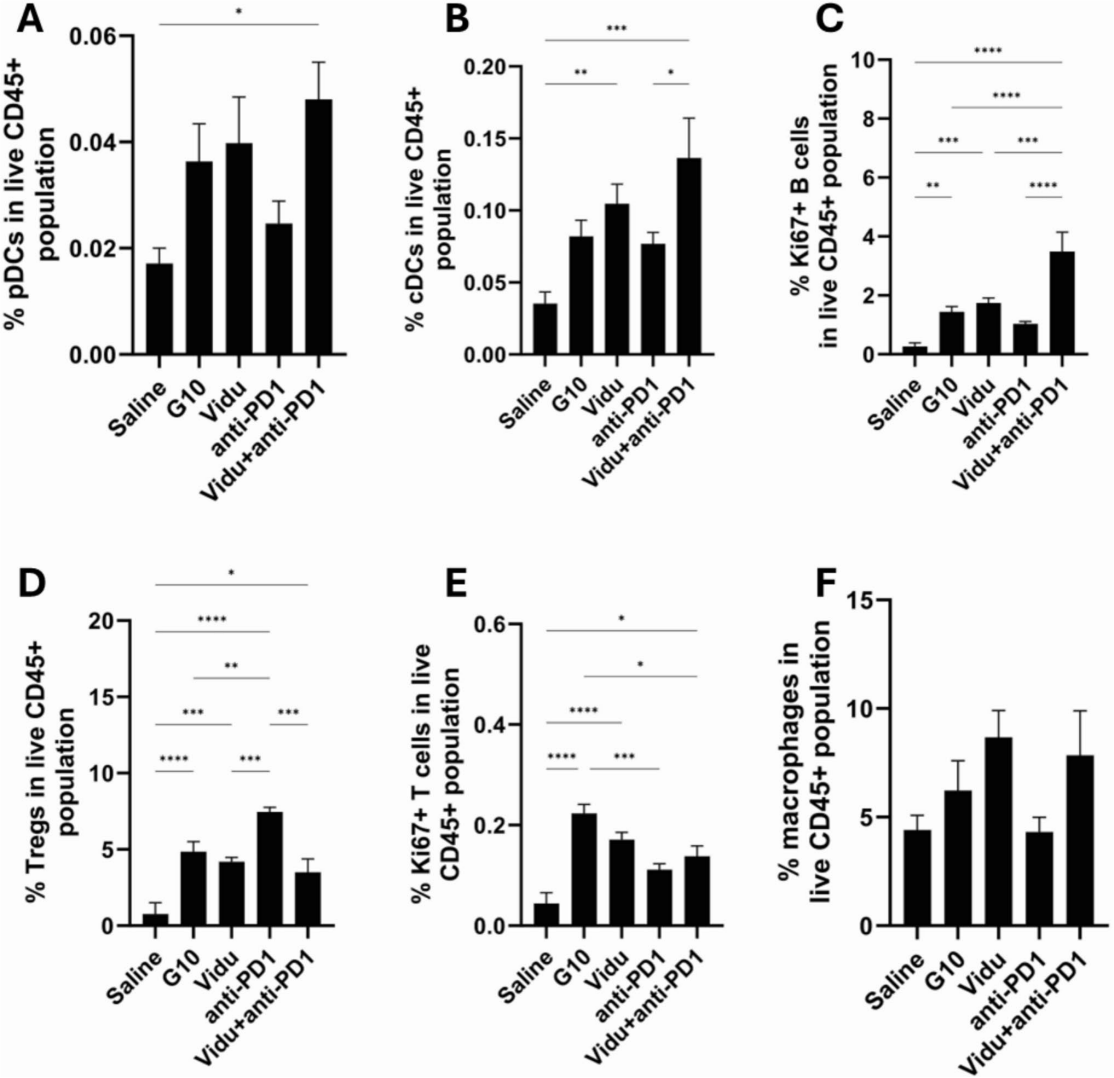
Immune cell levels in draining lymph node. Draining lymph nodes were harvested three days after the last indicated treatment. Cells were stained with relevant antibodies (*see methods Sect. 1.1.4*) and then analyzed by flow cytometry. The levels of **A** pDCs, **B** cDCs, **C** Ki-67 + B cells, **D** Tregs, **E** Ki-67 + CD8 + cells, and **F** macrophages in lymph nodes were assessed. Vidu: vidutolimod-treated group. One-way ANOVA was used for statistical analysis. The results are expressed as mean ± SEM. * *P* < 0.05, ** *P* < 0.01, *** *P* < 0.001, **** *P* < 0.0001

To characterize the immune cells infiltrating into draining lymph nodes proximal to the tongue, mandibular lymph nodes were collected four days after the last treatment (*see *Fig. 1* for treatment strategy*), stained for a range of immune phenotype-defining markers (*as described in Sect. 1.1.4*) and then analyzed by flow cytometry for percent subpopulations of CD45 + ve cells (CD45 being a marker for white blood cells/leucocytes). Vidutolimod + anti-PD1 had a significant effect in boosting pDC levels compared to the saline-treated group (*p* < 0.05), while vidutolimod alone showed a two-fold increase in pDC levels compared with the control but it was not significant (*p* = 0.14) (Fig. 7). Conventional DCs (cDCs) were found to be at significantly higher levels in the draining lymph nodes for mice treated with vidutolimod or vidutolimod + anti-PD1 compared to the control group. There was also a significant difference in levels of proliferating T cells and B cells between all treatment groups when compared to the control groups except for the anti-PD1-treated group. Although G10 alone can stimulate CD8 + T cells, its ability to activate DCs is not as strong as that of the combination of vidutolimod and anti-PD1. The increase in DC populations in the lymph nodes for the vidutolimod/vidutolimod + anti-PD1-treated groups likely reflects the ability of these treatments to efficiently stimulate TLR9 (since both cDCs and pDCs express TLR9 in mice) signaling and promote DC activation and encourage the extranodal DCs to migrate to the lymph nodes [37]. This suggests that such treatments have the potential to enhance antigen-presenting potency. This could have beneficial anticancer effects if tumor antigens are expressed at sufficient levels to prime adaptive immune responses.

### Effect of treatment on circulating cytokines

**Fig. 8:**
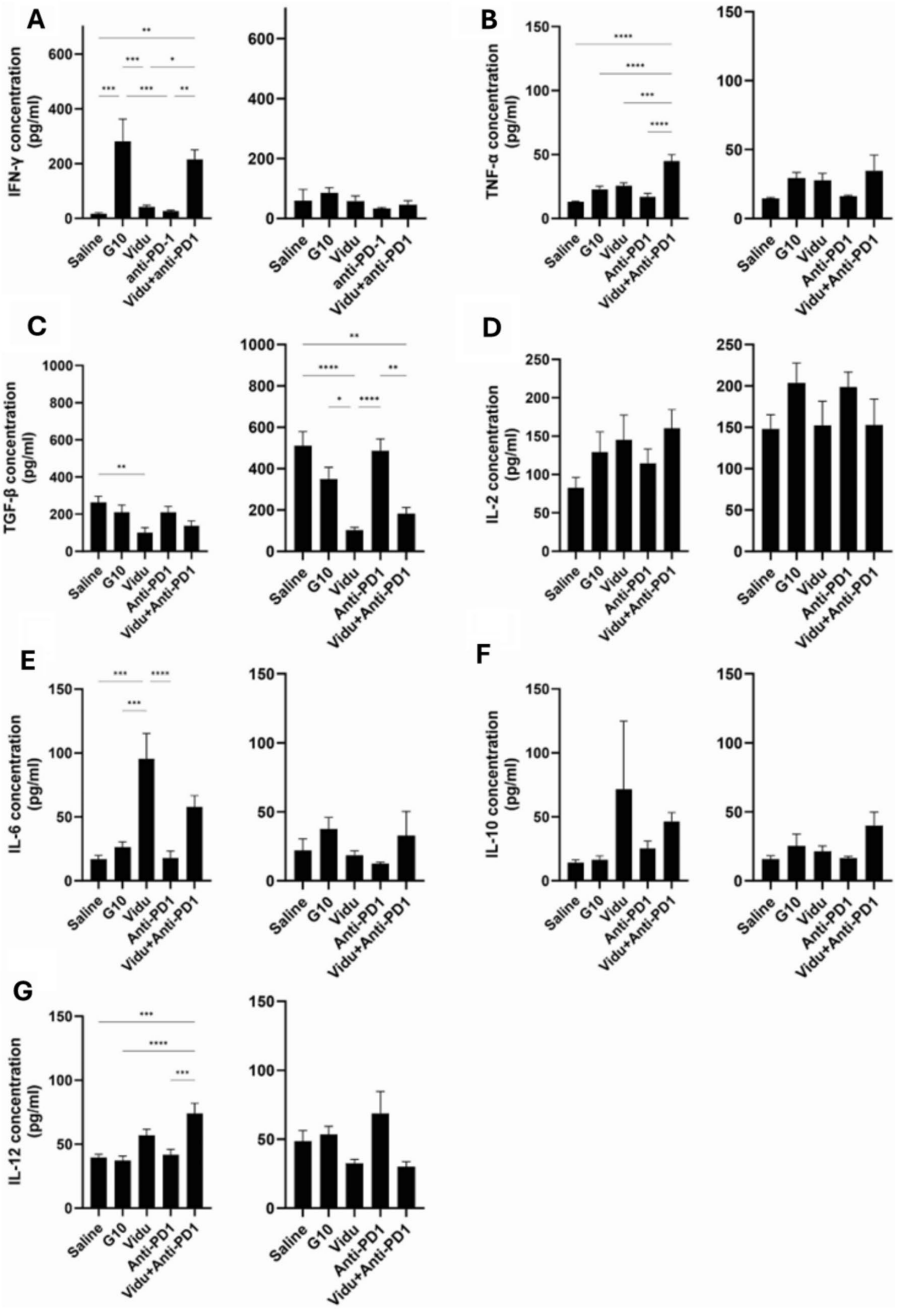
Cytokine release 24 h after first treatment (left) and third treatment (right). Concentration (pg/mL) of IFN-γ **A**, TNF-α **B**, TGF-β **C**, IL-2 **D**, IL-6 **E**, IL-10 **F**, and IL-12(p70) **G** was determined by a murine cytokine multiplex assay. Vidu: vidutolimod. One-way ANOVA was used for statistical analysis. The results are expressed as mean ± SEM. * *P* < 0.05, ** *P* < 0.01, *** *P* < 0.001, **** *P* < 0.0001

Mouse serum was collected 24 h after first treatment and third treatment for analysis of circulating cytokine levels. Significant increases were found for IFN-γ, TNF-α, and IL-12 24 hours after the first treatment of vidutolimod + anti-PD1 (Fig. 8. A, B, G). TNFα is a cytokine that can promote CD8 + T cell-mediated responses [38, 39], while IL-12 is also a promoter of CD8 + T cell expansion and function [40]. Thus, the combination of vidutolimod + anti-PD1 could prove to be a benefit in terms of eliminating tumor cells and creating tumor antigen for further CD8 + T cell activation as well as expanding and activating the tumor-specific CD8 + T cells already present in the diseased regions (prior to treatment). Vidutolimod alone showed a significant increase in IL-6 levels compared with the control (*p* < 0.001); and while a substantial (five-fold) increase was also seen for the vidutolimod + anti-PD1 group, it was not significant (*p* = 0.095). The elevated levels of IL-6, IL-12, and TNF-α in serum matched with observed increases in DC infiltration into lymph nodes. It suggests that vidutolimod + anti-PD1 together can enhance pDC activity and potentially contribute to a more robust immune response against antigenic tumors (*discussed further below*). A sustained and significant drop in TGF-β1 was found in the vidutolimod-treated group versus the control as observed after both the first and third treatments (Fig. 8). It indicated that vidutolimod treatment may promote decreased immune suppression and therefore promote an environment more favorable to support immune-mediated anticancer activity due to TGF-β1’s immunosuppressive properties. TGF-β1 is known to play a complicated role in a variety of cancers where it tends to be tumor suppressive during the early stages of tumor development and tumor promoting during late-stage tumor development, thus complicating an interpretation of its role in this model where a combination of mild and severe oral dysplasia was treated.

Further studies are required where the safety of the combination treatment is established, and the efficacy and mechanisms of action are more clearly defined. For instance, allowing the mice further time posttreatment (i.e., longer than 16 days) to be monitored for regression of lesions since activation of naïve CD8 + T cells may take longer than 16 days to show significant changes in disease severity. Since a limitation of this study was that the oral dysplasia model relied on qualitative rather than quantitative changes as assessments of disease status, circumstances that result in more stark differences in phenotype may be required; as mentioned this may be achieved by monitoring for longer, and also extending the 4NQO treatment period in order to create a higher percentage of mice with symptoms of severe oral dysplasia.

## Conclusions

The treatments employed in this study aimed to curb the progression of oral dysplasia or at the very least create an immune environment that potentially favored an immune-based anti-oral dysplasia response; and we found that our combination treatment (vidutolimod + anti-PD1) was the most promising in this regard as evidenced by the resulting significant changes in expression of immune markers and cytokines at certain points during the therapy. The levels of pDCs and Ki-67 + CD8 + T cells increased significantly after vidutolimod + anti-PD1 therapy as did the TNF-α and IFN-γ serum levels. The enhancement in pDC and cDC populations, when vidutolimod was used with anti-PD1, suggests that the combination of these therapies might optimize the potency antigen-presenting cell responses (responsible for triggering CD8 + T cell activation/function) more effectively than either agent alone.

This study documented the change in immune parameters (cell populations and cytokines) in an oral dysplasia context progression under conditions after treatment with different defined immune therapies in terms of both local and systemic immune responses. The findings suggest the importance of a strategic combination of vidutolimod and ICB in managing and potentially reversing high-risk precancerous conditions. Further studies could expand on these results to optimize treatment schedules and dosages, and enhance therapeutic outcomes in clinical settings.

## Data Availability

No datasets were generated or analyzed during the current study.
